# EUPRO - A reference database on project-based R&D collaboration networks

**DOI:** 10.1038/s41597-024-03129-y

**Published:** 2024-03-12

**Authors:** Thomas Scherngell, Michael Barber, Georg Zahradnik, Anna Wolfmayr, Xheneta Bilalli Shkodra

**Affiliations:** Austrian Institute of Technology, Vienna, Austria

**Keywords:** Funding, Economics, Databases

## Abstract

The EUPRO database enables the analysis of participation patterns of organisations in and across different European R&D funding initiatives and the investigation of resulting collaborative R&D network structures and dynamics. The perimeter of EUPRO is currently more than 600,000 R&D projects funded by European (EU, transnational or national) research funding organisations, comprising systematic information about contents of the R&D projects, their participating organizations (including organisation type and location), and a number of additional characteristics (e.g. underlying policy instrument and programme). This scientific data descriptor serves as illustrative information source for users, both from science as well as from policy. It discusses the conceptual background and derives respective analytical opportunities for different actual, highly relevant debates in innovation studies and related fields. Moreover, the data collection process is described in a compact manner, as well as how the collected data are harmonized and aggregated into a suitable data model for analytical purposes. Finally, we put forward issues of technical validation, data quality and enrichment, and usage notes on how to access EUPRO.

## Background & Summary

Nowadays, we can observe a strong consensus that innovation is driven by knowledge creation in a web of collaborating organizations of different types, and at different geographical locations^1,2^, often referred to as innovation or R&D collaboration networks. In the scientific debate, such arrangements of collaborative R&D are usually described by the notion of R&D collaborations, or R&D collaboration networks in a wider sense^3^. The need for collaboration arises from the specific features of new knowledge, especially its tacit and highly specific content. The increase of collaboration activities in recent decades stems to a large extent from the rising costs and risks associated with R&D, and the ongoing trend towards more complex knowledge production due to the conversion of disciplines in previously separate fields^4^, also referred to as converging technologies. Rising costs of R&D motivate network participation by the mobilization of synergies between the collaborating actors and the easier reach of a critical mass of the new knowledge, while the increased complexity of R&D stimulates complementary networks where participating partners benefit from each other by mutual learning and the exchange of complementary knowledge. In practice, both synergic and complementary functions of networks often overlap.

The new focus on R&D collaboration has also been reflected by significant efforts at the European level to support them by public funding. For instance, the primary European innovation policy instrument, the EU Framework Programme (FP), intends to foster collaborative R&D activities across countries and regions. The FP has been designed specifically to pool resources and foster international pre-competitive R&D collaborations by intensifying interactions among researchers and regions. By means of these instruments the EU has co-funded thousands of transnational collaboration projects since its implementation back in 1984 (see^5^, among others). But also at national funding, we have seen an enormous increase of project-based and collaborative funding instruments.

Against this background, the empirical investigation of publicly funded R&D collaboration networks, in particular their dynamics, i.e. how they evolve over time, in technological and in geographical space, has attracted a great deal of attention in the past two decades from a scientific and a policy perspective (e.g. see^3^ for an overview). Collaborative R&D projects involve a clear research focus and time horizon as well as certain conditions on the geographical range of partners, giving rise to networks of innovating and researching organizations stimulating the flow of knowledge between these partners, but also diffuses to other actors located in certain geographical areas where such partnerships take place.

The EUPRO database focuses on this type of networks and has been designed from its very beginning back in 2005 to enable novel empirical research in this direction. It has meanwhile become a reference dataset^6^ for the empirical observation of publicly funded R&D collaboration networks of different types across Europe, also as important part of the RISIS research infrastructure (risis2.eu, see details in the section that follows). In essence, EUPRO comprises information on R&D projects and all participating organizations funded by different public R&D funding programmes in Europe, organized at EU level (mainly the EU FP, but also COST, EUREKA and JTIs) or by individual countries (national R&D funding channels). EUPRO is maintained and regularly updated, and also constantly advanced by additional modules, most importantly the addition of national programmes next to European and transnational ones. In its recent deepening activities, EUPRO has extended its focus to collect data on R&D projects funded at national levels, the so called NATPRO module.

### An overview of EUPRO

**Fig. 1:**
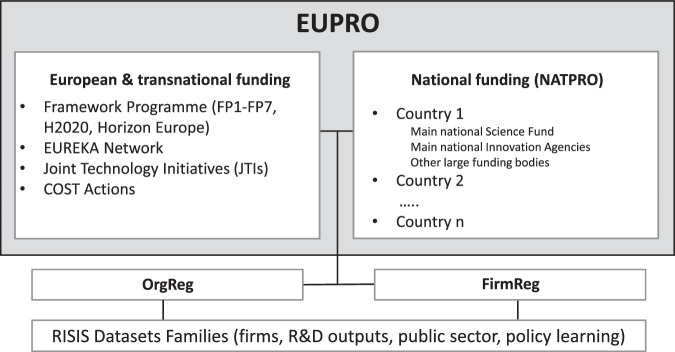
EUPRO coverage and embedding.

Figure 1 provides a schematic illustration on the vision of EUPRO comprising an umbrella for systematic and cleaned information on project-based R&D projects and collaboration at different spatial levels. In essence, EUPRO comprises information on R&D projects and all participating organizations funded by different public R&D funding programmes, involving European and national programmes of European countries. The included EU Framework Programmes (FP1-FP7, H2020, Horizon Europe) are by far the most important EU funding programmes for research and innovation, with a budget of 95.5 billion Euro for the ongoing Horizon Europe programme^7^ and are complimented with other smaller and more specific European and transnational funding programmes (EUREKA, JTIs and COST Actions). In respect of national funding progammes, data for the main national funding organization for basic research (national research council) is included and complimented with data from some sectoral research funding agencies as well as national innovation agencies if available. EUPRO has been used over the past ten years for research studies but also in contract research for national and international customers, such as the European Commission. The database is maintained and regularly updated (annual additions of new information), and also constantly advanced by additional modules.

For the funding programmes part of EUPRO, it basically covers information on:

- **projects** (such as project objectives and achievements, project costs, total funding, start and end date, contract type, information on the call), and
- **participations** (standardized name of the participating organization, contact person with contact details, organisation type, and geographical location)

Next to the coverage of EUPRO, Fig. 1 also points to its embedding in the RISIS research infrastructure (risis2.eu), and by this, its connection to other datasets relevant for innovation and policy studies, such as publication or patent dataset. RISIS is a European Research Infrastructure for Science, Technology and Innovation Policy Studies, freely accessible for research, providing data and indicators about research and innovation activities. Currently, 15 datasets are open and accessible (https://www.risis2.eu/risis-datasets) covering topics like R&D and innovation outputs (patents, publications, trademarks, and R&D projects as covered by EUPRO), firm innovation (startups, fast growing firms, large R&D companies), public sector research (higher education institutions, research careers) and innovation policy learning (policy instruments and evaluations). The potential of connecting EUPRO with these datasets has been realized by two organization registers developed within RISIS, the so-called OrgReg facility (a register for public sector research organizations) and the FirmReg, a register for European firms. Via these two registers, EUPRO can be linked at organizational level to other datasets within RISIS, enabling to address completely new issues like e.g. the direct relation of R&D projects (from EUPRO) to specific research (publication) or inventive (patents) outputs.

The embedding in RISIS implies some important consequences for data treatment, e.g. name standardization of participating organizations to projects, that are also discussed in this descriptor (see the section on technical validation). In terms of its magnitude, Table 1 gives on overview on the EUPRO perimeter in 2023, separately for the most important European funding programmes, and jointly for NATPRO denoting the collection of all national funding programmes (currently 16 countries included). Due to the large number of countries included in the NATPRO, the national module contains the highest number of projects. In fact, 81% of all projects within EUPRO are nationally funded. Within the European and transnational funding module, 94% are projects of the framework programmes.

**Table 1:** EUPRO perimeter 2023.

Programme/Module			Period	Projects	Participations
*European & transnational funding*	*Framework Programmes*	FP1	1984–1987	3,348	7,972
		FP2	1987–1991	3,987	19,184
		FP3	1990–1994	5,474	30,768
		FP4	1994–1998	14,524	67,831
		FP5	1998–2002	16,026	78,562
		FP6	2002–2006	10,100	75,356
		FP7	2007–2013	25,785	140,048
		H2020	2014–2020	35,378	173,084
		Horizon Europe	2018–2021	5,763	40,274
		*Subtotal*	1984–2021	*120,385 (18%)*	*633,079 (43%)*
	*Other*	EUREKA^1^	1985–2019	5,932	26,233
		JTIs^2^	2008–2014	133	2,612
		COST	1971–2014	1,132	35,543
		*Subtotal*	1971–2019	*7,197 (1%)*	*64,388 (4%)*
		*Subtotal*	1971–2021	*127,582 (19%)*	*697,467 (47%)*
*National funding (NATPRO*^3^			2010–2022	*528,693 (81%)*	*786,138 (53%)*
**Total**			**1971–2022**	**656,275 (100%)**	**1,483,605 (100%)**

Note: ^1^including all projects starting before January 2020, ^2^Including ARTEMIS (calls 2009–2013), ENIAC (calls 2008–2013), ECSEL (2014); ^3^NATPRO includes 16 countries and 78 RFOs. Time coverage up to 2022 for 6 countries and up to 2020 or 2021 for another 9 countries (Italy’s data is only available until 2015). For 11 NATPRO countries data before 2010 is also included.

### EUPRO Usage in scholarly literature and projects for policy

The EUPRO database has emerged over the past decade as a pivotal resource in the exploration of project-based and publicly funded R&D networks within the broader research domain investigating structures and dynamics of R&D collaboration networks (see^3^ for an overview). A comprehensive review of scholarly literature reveals the dataset’s significant impact, with over 150 documents, including more than 40 journal articles, employing EUPRO data (retrieved from google scholar as of November 2023). These works leverage EUPRO to delve into the structure, dynamics, and effects of project-based R&D collaboration networks on knowledge production and innovation. This is especially relevant for elucidating the evolution of the European Research Area (ERA).

Within the literature stream exploring R&D collaboration networks, the application of the EUPRO dataset spans several domains, illustrated by specific subfields:

i. **Drivers for the establishment of R&D collaboration networks**: The first subfield uses EUPRO to shed some light on drivers that affect the formation of such networks. This is often done at the regional level of analysis, shifting attention to the estimation of barriers for cross-regional R&D collaboration, which depend on different types of separation effects, such as geographical, technological or cognitive distance. The study of Barber and Scherngell^8^ demonstrates in this context that European R&D networks are not homogeneous and show distinct substructures characterized by spatially heterogeneous community groups. The studies of Scherngell and Lata^9^ and Lata *et al*.^10^ focus on the evolution of such separation effects over time, showing that geographical barriers decrease but stay important. Neuländtner and Scherngell^11^ extend this approach by specifically comparing geographical to network structural effects. Finally, Wanzenböck *et al*.^12,13^ identify key drivers of regional involvement in European R&D collaboration networks including factors like the existing scientific and technological capacities of the regions.
ii. **Impacts of R&D collaboration networks**: This subfield mobilizes EUPRO to estimate how R&D collaboration networks affect knowledge production and innovation. Hoekman *et al*.^5^ demonstrate that R&D collaboration networks significantly stimulate subsequent co-publication activities between pairs of EU regions. This particularly benefits lagging regions by enhancing their integration into the broader European research community and facilitating knowledge exchange. Wanzenböck and Piribauer^14^ provide evidence that increasing embeddedness in EU funded R&D networks leads to positive immediate impacts on regional knowledge production, and that regions with lower levels of own knowledge endowments more likely exploit the positive effects. Uhlbach *et al*.^15^ indicate that participations in R&D networks have a positive effect on the development of new specialisations of regions. Lastly, Neuländtner and Scherngell^16^ show that the embedding in inter-regional R&D collaboration networks is a significant driver for both explorative and exploitative modes of knowledge creation.
iii. **Description of participation patterns in R&D collaboration networks**: This subfield refers more to descriptive works aiming to characterize structures and dynamics of R&D collaboration networks (often focused on different topics and/or geographical spaces), or to describe participation patterns to networks (mainly the FP) of specific organization types (e.g. firms or universities). Exemplifying studies in this context are Lepori *et al*.^17^ analysing patterns of participation of higher education institutions (HEIs) in the EU-FP, or Villard *et al*.^18^ investigating the participation patterns in R&D collaboration networks of the EU-FP in nanoscience and technology.

Regarding the thematic foci, it is worth mentioning that scholarly works using EUPRO mobilize very different quantitative methods. Most recently, the study of Ancona^6^ uses EUPRO for testing and illustrating a novel methodology to disambiguate organization names This is not only of interest in terms of content, but also shows whether the setting of EUPRO (e.g. geographical and time coverage) and its quality makes it eligible for advanced quantitative methods and analyses to be employed. Another element that has become specifically salient in these recent scholarly usages of EUPRO is the increase of joint applications with other datasets (also in particular within the RISIS framework). Specifically important becomes the combination of EUPRO with other R&D output-oriented datasets, e.g., on patents and publications (see e.g. Neuländtner and Scherngell^16^) that are directly linkable via RISIS identifiers. Another example in that direction constitutes the joint integration of indicators from EUPRO together with other datasets in the RISIS-KNOWMAK tool (knowmak.eu) for monitoring R&D activities in Europe at a very fine-grained topical level.

## Methods

EUPRO is based on a systematic collection of secondary data from different data sources. Availability of this source data in terms of access condition, coverage and completeness is regularly monitored. Note that no primary data are collected. The sources used are all subject to open science principles. At the European level, CORDIS is clearly one of the main sources given the importance of EU-FP projects within EUPRO. At national level, data are collected from national R&D funding organizations, or from national information systems.

### The data collection process

In general, EUPRO relies exclusively on public data, available from different sources, in different formats and via different access channels, in correspondence with respective access and usage regulations (e.g. only for research purposes). Project data for the different components of EUPRO have been collected depending on the data availability either via download or via web scraping by automatically extracting and structuring information from various XML-Files. Table 2 provides an overview on the collection process of the different modules. In case of the European Framework Programme (FP), project data included in the current version of EUPRO for FP7 and H2020 were downloaded from the CORDIS project database^19,20^ and are updated yearly. On the contrary, FP projects collected earlier (FP1 to FP6) were web scraped from the CORDIS website in March, 2020 (https://cordis.europa.eu/). Meanwhile data on these projects are also available as download in xlsx format^21–26^. Horizon Europe projects which will be included in the updated version of the EUPRO were also downloaded from the CORDIS project database^27^ recently (but are still in processing).

**Table 2:** Overview EUPRO data collection.

Module	Download from National Research Information Systems (NRIS)	Download from individual RFO websites	Download from OpenAire EXPLORE	Webscraping
European & transnational funding modules	—	FP7^19^, H2020^20^, HEU^27^, JTIs	—	FP4^21^, FP5^22^, FP6^23^, FP1^24^, FP2^25^, FP3^26^, Eureka, COST
National funding (NATPRO)	France^31^, Estonia^32^, United Kingdom (programme and project data)^33^, Poland^34^, Czechia^35^, Slovenia (restricted download)^36^, Slovakia^37^, Lithuania^38^, Sweden^39^	Austria^40,41^, Ireland^42^, Switzerland^43^	Croatia^44^, Finland^44^	Germany^45^, United Kingdom (participation data)^33^, Italy^46^

Web scraping was also utilized to access EUREKA and COST actions projects. Two data sources were accessed for the collection of EUREKA project data. First, for projects starting between 1985 and 2014 the EUREKA website (http://www.eurekanetwork.org) was exploited in January 2017. Second, projects starting after 2014 were retrieved from the interactive dashboard (https://www.eurekanetwork.org/about-us/interactive-dashboard) in April 2021. Raw project data for COST actions were web scraped in May 2014 using the COST website (https://www.cost.eu). Similar to the newest framework programmes, project data for the three JTIs ARTEMIS, ENIAC and EXCEL were downloaded from the respective research funding organizations’ websites in May 2017 (https://www.artemis-ju.eu; http://www.eniac.eu; https://www.ecsel.eu).

As for the NATPRO module, most raw data for national projects were retrieved from central registries of public funded R&D projects, i.e. National Research Information Systems (NRIS), providing project data for the main RFOs (see Table 2). Except for Slovenia, the data was freely available. If no central registry existed or the data on NRIS was not downloadable or had insufficient data available, either OpenAIRE (OpenAIRE – Explore. n.d.), an open research search portal, or individual RFO websites were accessed to download national project data. For Germany and Italy, data had to be extracted through web scraping from the national RFO websites which had sufficient data available. Data for the United Kingdom was collected using both collection methods. While there was data available from a NRIS both for projects and collaborating organizations, the two information sets had to be linked using information extracted through web scraping. If readily available, data on project information were collected in English, otherwise, they were included in national language. The accessibility of English variables in the NATPRO module, thus, depends on the country and the variable.

Data collection involved using web scraping, a method for extracting information from websites. For the earlier versions of the EUPRO database, specifically FP1-FP6, EUREKA and COST, the programming language *Ruby* (www.ruby-lang.org) was the primary tool of choice. Its associated libraries such as *Watir*, *Nokogiri*, and *Mechanize* were utilized to navigate and extract data from HTML and XML, as well as automate web browser interactions. In the case of extracting data for the NATPRO module, namely for IT and DE, *Python* was the programming language of choice. Parsing of HTML content was accomplished using *Beautiful Soup*, while *Selenium* was used for conducting automated tests.

Websites typically adhere to a consistent page structure, with special cases that the scraper attempts to handle to the best. This uniformity across pages facilitates data extraction by the scraper. To navigate through these pages effectively, a strategy is needed to determine the starting point and the process for moving through the pages. The scraped data is typically stored in either CSV files or directly within a database (*MySQL*). The decision of how many distinct CSV files or database tables to use depends on the source data. The scraper also maintains logs to monitor any potential issues encountered during the scraping process. These logs serve the dual purpose of identifying instances where data may not have been successfully scraped and providing information about what and when was scraped. Once the scraping process is complete, the data is imported into a database for further utilization.

The EUPRO central database is stored in a Database Management System, such as Microsoft Access, which is suitable for non-technical users due to its file-based storage system. Additionally, the data is stored in a Relational Database Management System, such as MySQL, for more advanced applications, including the EUPRO Application Programming Interface (API), as explained in the Usage Notes section.

Coming from diverse sources with different goals, the data collected are not generally compatible between the components of EUPRO nor are they necessarily well suited to policy-relevant analyses. Further, the data will not generally be readily used in conjunction with related external data sources (e.g., data on publications or patents). To address this, a standardization process is undertaken, largely consisting of aligning the collected data with broadly accepted standards and definitions; creation of novel internal standards is avoided. The standardization process thus also partially serves as a form of technical validation, detailed later in the Technical Validation section.

## Data Records

The latest release of EUPRO is available at in the RISIS research infrastructure (risis2.eu). A static copy of the dataset at the time of review has also been uploaded to Figshare (figshare.com/articles/dataset/Projects_Participations_csv/24681258). EUPRO includes four modules on European & transnational funding (EU-FP, EUREKA, JTIs, COST) and a module on national funding (including 16 submodules for each country included). Additional modules (transnational funding) or submodules (additional countries) are constantly added to EUPRO. These additions reflect the changing needs of data users and the increasing availability of publicly source data, in particular on the national level.

### Variables and data structure

**Fig. 2:**
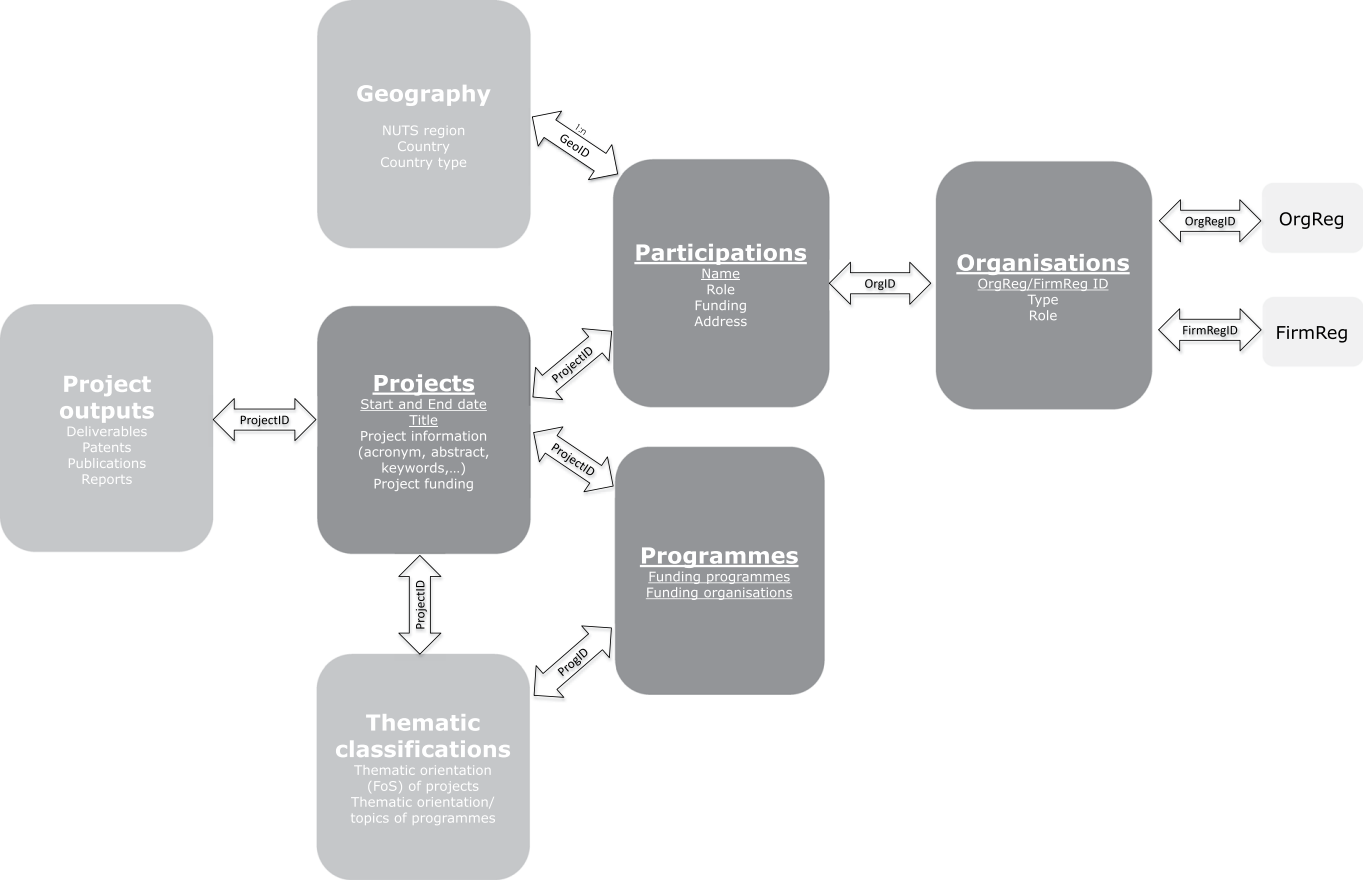
EUPRO basic structure.

The EUPRO variables are grouped into the four main categories projects, organizations, participations and programmes with additional variables on project outputs, thematic classifications (on the level of projects and programmes) and geography. Figure 2 below provides an overview of the basic structure of the EUPRO modules and linkages to external organization registers (www.risis2.eu/risis-organisation-registers), namely OrgReg (a reference database on public research) and FirmReg (a reference register on private actors), enabling actor-level harmonisation at European level.

Table 3 provides and overview of the data coverage for each main variable per EUPRO module. The main EUPRO variables include:

- Information on the level of R&D projects, such as start and end date, project title, project acronym, abstract, keywords but also project funding and/or costs
- Information on the level of the participations for each project such as name of the participating organization, role (coordinator or participant) and funding per participant.
- Information on the level of the participating organisations such as type (Industry, Higher Education Inst., Research Organisations, etc) and and linkages to external organisation registers providing additional organisation level information (including demographic information, a set of characteristics of the entities and a set of geographical locations).
- Information on the level of the programmes including the funding organisation and funding country.
- Information the project outputs including deliverables, patents, reports and publications.
- Thematic classifications on the level of projects broken down by Key Enabling Technologies (KETs, six classes and 42 subclasses) and Sustainable Development Goals (SDGs, 15 classes and 61 subclasses).
- Thematic classification for programmes based on Fields of Science or linking with other RISIS datasets.
- Information on geography of the participation including the country and the region (for the NATPRO module via linking with the external organization registers OrgReg and FirmReg). The geographical location corresponds to the address at the time the project was funded. It represents where the participation took place, so it is not updated in the event organizations that relocate or end operations.

For the most recent and comprehensive description of specific variable definitions, types, data sources and the entity relationship models used, please refer to the latest version of the technical documentation of the respective EUPRO module.

**Table 3:** EUPRO main variables and coverage per module.

Categories	Main variables	FP1-FP7, H02020 and Horizon Europe	Other trans-national funding (EUREKA, JTIs, COST)	NATPRO
Projects	ID (unique identifier)	+	+	+
	Start and End date	+	+	+
	Title	+	+	+
	Acronym	+	+	o
	Call	+	o	o
	Abstract	+	o	o
	Keywords	+	o	o
	Costs/Funding	+	+	o
Participations	Name	+	+	+
	Role (coordinator or participant)	+	+	o
	Funding	+	o	o
	Address	+	o	o
Organizations	Name	+	+	+
	Type (Industry, Higher Education Inst., Research Organizations, etc)	+	o	via Link to OrgReg/FirmReg
	OrgReg ID	+	−	+
	FirmReg ID	o	−	o
Programmes	Name	+	+	+
	Funding Country	+	+	+
	RFO name	+	+	+
	RFO acronym	+	+	+
Project Outputs	Deliverables	+	−	−
	Publications	+	−	−
	Patents	+	−	−
	Reports	+	−	−
Thematic classifications	Thematic orientation (KETs, SDGs, FoS) of projects	+	−	o
	Thematic orientation of programmes	+	−	o
Geography	NUTS3	+	+	via Link to OrgReg/FirmReg
	Country	+	+	

Note:+included; o partially included; − not included.

## Technical Validation

The components of EUPRO all have seen substantial efforts to improve the quality of the raw data. The principal goal of these efforts is to improve the suitability of the data for research purposes, but they as well provide an opportunity for detection and correction of data errors. For example, assigning NUTS3 regions to FP project participants makes use of detailed address information, which may reveal errors in the country code and allow for a manual correction to be made.

Data quality was improved using a multi-faceted approach. Steps taken for quality improvement that directly allowed for validation include (i) standardizing country codes and (ii) regionalizing using address information.

(i) Standardizing the country codes is a manual process. Its most basic aspect is conversion of country information in the raw data to the ISO 3166-1 alpha-2 format. Along with detecting errors in the raw country information, this step also avoids common issues with the codes used for certain countries, e.g., whether the United Kingdom is represented as UK or GB. Using a standard code can also reveal more subtle interoperability issues, such as the handling of disputed territories or dependent territories.
(ii) Regionalization uses a two-stage process. The first stage uses a mapping from postal codes to NUTS3 regions. Where that is unsuccessful, address information is used to determine corresponding latitude and longitude by making use of online geocoding tools. These geographical coordinates in turn can be assigned to NUTS3 regions by comparison with Eurostat-provided polygons defining the regions^28^. Because regionalization uses more detailed geographical information, it provides further opportunities for data validation in the form of inconsistent countries and regions. Any discrepancies are manually corrected. Note that the geographical location for participations is based on the values provided by the project partners when the projects was funded. This is not updated with changes and thus reflects where the project participation took place. Geographical information is also provided for organizations at the country level. This corresponds to the legal seat of the. These values can be updated, but any such changes will only appear in EUPRO with later releases.
  Additional data improvements were made for purposes of interoperability, enriching the EUPRO components with standardized terminologies to allow data on project funding to be used in conjunction with, e.g., data on patents or publications. These steps indirectly allow data validation by comparing against other sources of data. Relevant data enrichment steps include (iii) linking organization names to the OrgReg and FirmReg organizational registers and (iv) classification of projects as relevant to particular Sustainable Development Goals (SDGs).
(iii) Harmonization of organization names with the OrgReg and FirmReg is ultimately a manual process but supported with recommendation algorithms to speed the process (inspired by Raffo and Lhuillery^29^). These algorithms are based on statistical properties of the organization names, chiefly the frequency of adjacent characters in the names. Similar organization names can then be ranked, allowing manual review efforts to focus on the most likely candidates and speeding the overall process. The review process provides a significant opportunity to observe problems in the organization names. As well, an additional validation step is provided by computing the number of project participations per organization and reviewing to ensure that key research actors are present.
(iv) Association of projects with the EU SDGs is executed using a classifier service built on the GATE system (gate.ac.uk/projects/knowmak). GATE uses a natural language processing approach to make the SDG assignments, which is based on assessment of the descriptive text for projects. As with the harmonization with the organization registers, the SDGs allow a validation step by computing the geographical distribution of project participations within specific SDGs, with comparison of the results for regions with known thematic foci relevant to the SDGs.

## Usage Notes

The basic version of EUPRO is publicly available and has been uploaded to the figshare repository (figshare.com/articles/dataset/Projects_Participations_csv/24681258)^30^. The full-fledged version including links to other datasets can be accessed via the RISIS research infrastructure (risis2.eu) under controlled access for research purposes. As described in the RISIS Code of Conduct (available under rcf.risis.io/access-request/datasets), controlled access defines that the researcher entering RISIS has to clearly outline a research path by a description of the research purpose. Applications for access can be done upon registration under rcf.risis.io/access-request/new, comprehensive documentations are provided in the open RISIS zenodo community (zenodo.org/communities/risis), instructions and guidelines on what is included how to use the RCF web application are available in the application documentation (docs.risis.io/gettingstarted/introduction). It is excluded from commercial usage in relation to the legal conditions from most source data providers (such as for instance CORDIS as the source data provider for the FP module of EUPRO). Downloaded data can be analyzed using any software tools suitable for analysis needed. Given the focus on networks, software packages like Gephi, or network libraries in R or python are specifically useful.

The EUPRO Application Programming Interface (API) has been developed to facilitate the transfer of data from EUPRO central database to other data platforms that can be used for data extraction and analysis. This API offers a range of endpoints, allowing users to access the complete dataset as well as filtered data based on criteria such as year range, geographical parameters, or specific keywords found within project descriptions. The implementation was done using the PHP programing language within the Laravel (https://laravel.com) Framework and it is documented in standardized API documentation, such as Swagger (https://swagger.io). The API provides data in a JSON format, which aligns perfectly with the structured nature of databases like EUPRO.

## Data Availability

AIT has developed the web scraping script for data collection of parts of EUPRO; it can be retrieved from: https://figshare.com/articles/software/cordis_wrapper_rb/24711738.
